# Detection of Adulteration in Canola Oil by Using GC-IMS and Chemometric Analysis

**DOI:** 10.1155/2018/3160265

**Published:** 2018-09-23

**Authors:** Tong Chen, Xinyu Chen, Daoli Lu, Bin Chen

**Affiliations:** School of Food and Biological Engineering, Jiangsu University, Zhenjiang 212013, China

## Abstract

The aim of the present study was to detect adulteration of canola oil with other vegetable oils such as sunflower, soybean, and peanut oils and to build models for predicting the content of adulterant oil in canola oil. In this work, 147 adulterated samples were detected by gas chromatography-ion mobility spectrometry (GC-IMS) and chemometric analysis, and two methods of feature extraction, histogram of oriented gradient (HOG) and multiway principal component analysis (MPCA), were combined to pretreat the data set. The results evaluated by canonical discriminant analysis (CDA) algorithm indicated that the HOG-MPCA-CDA model was feasible to discriminate the canola oil adulterated with other oils and to precisely classify different levels of each adulterant oil. Partial least square analysis (PLS) was used to build prediction models for adulterant oil level in canola oil. The model built by PLS was proven to be effective and precise for predicting adulteration with good regression (R^2^>0.95) and low errors (RMSE ≤ 3.23).

## 1. Introduction

Canola oil is a vegetable oil derived from rapeseed which has low erucic acid content [1]. In the 1980s, in order to decrease the health concerns about erucic acid, rapeseed varieties free from erucic acid were developed by using selective breeding [2, 3]. And then, these varieties were called canola. Nowadays, China has become one of the major consumers of canola oil, and it is also popular with consumers in Canada, Europe, and South America. Canola oil can provide consumers with many health benefits that others cannot provide. For example, canola oil is low in saturated fat and high in polyunsaturated fats, with a good ratio of omega-6 to omega-3, which make it very suitable for cooking [4–6]. Since canola oil has its specific function to human body, it has become one of the most susceptible food materials adulterated with other vegetable oils of lower quality, which is a serious threat to the health of consumers. Therefore, it requires reliable tools and methods for analyzing the purity of edible vegetable oil.

Many techniques have been developed and used to detect adulteration in oil. These techniques include physical-chemical analysis, spectral analysis, gas chromatography (GC), gas chromatography-mass spectrometer (GC-MS), and electronic nose. Physical and chemical analysis includes sensory evaluation, colorimetry, centrifugation, and freezing. These traditional methods are simple and convenient and suitable for local monitoring. However, physical and chemical analysis methods are not accurate, require high degree technical expertise, and can only determine whether the sample is adulterated without finding out which specific component is adulterated. Spectral methods, e.g., Nuclear Magnetic Resonance Spectroscopy (NMR) [7], Raman [8], Fourier Transform Infrared (FTIR) [9], and Fluorescence [10], were shown to be useful for detection and quantification of adulteration in oil. However, their data analysis requires specialized software and complex algorithms which are difficult for common users to master. Chromatographic methods, such as GC-FID (flame ionization detector) [11], GC-MS [12], and high performance liquid chromatography (HPLC) [13], have been proven to be effective in detecting adulteration in oil. Nevertheless, the requirement for standard samples and high input of time and labor make them unsuitable for on-site analysis, thus limiting the wide use of them. Electronic nose [14] also has been used to evaluate the quality of oil. However, it needs electrode activation process during which sensor poisoning may occur depending on operation and ambient conditions.

Ion mobility spectrometry (IMS) is an analytical technique and was initially developed for the detection of explosives and chemical warfare agents [15]. At present, it has been widely used in novel application in the field of agricultural products and foods [16]. IMS is used to separate and identify ionized molecules in the gas phase based on their mobility in a carrier gas, which is considered as a screening technique due to its ability to identify the properties of samples at considerable low cost and short analysis time without pretreatment. On the other hand, IMS has limitations in detecting complex sample (e.g., food) for having low resolution and the risk of mutual interferences between analytes [17]. Yet, if combined with GC, the capability of IMS in separating various components is strengthened. Chromatographic elution of each target compound can be automatically analyzed and the obtained data information is richer because both retention time and drift time information are included [18]. GC-IMS has been shown to be able to characterize and discriminate adulteration in oil, wines, honey, and meat [19–23]. Successful applications have been reported on the determination of aldehydes in oil, adulteration in extra virgin olive oils by using UV-IMS [24], and determination of volatile compounds [25].

Most of the previous reports on canola oil analysis mainly focused on the adulteration detection and main component quantification of oil species [8, 26], with few studies performed on aroma differentiation. Odour is an important quality criterion for edible vegetable oil. The previous relevant studies often transformed the matrix to vector (like peaks selected manually as variables) for chemometric analysis by UV-IMS or GC-IMS, which may result in losing information of certain analytes. In addition, to the best of our knowledge, no recent work has been conducted using chemometrics for feature extraction of the two-dimensional data produced from GC-IMS instrument. Therefore, the potential use of GC-IMS for detection of canola oil adulteration was investigated. The aims of this study were (1) to investigate the use of GC-IMS combined with pattern recognition methods to detect the presence of adulterant in canola oil, (2) to apply a new method to extract information for two-dimensional data, (3) to build a model for content prediction of adulterated oil in canola oil, and (4) to develop a rapid method for adulteration detection in canola oil.

## 2. Materials and Methods

### 2.1. Preparation of Oil Blends

3 canola oil samples were provided by Ningbo Entry-Exit Inspection and Quarantine Bureau (Zhejiang Province, China), while sunflower oil (2 samples), soybean oil (3 samples), and peanut oil (2 samples) were all purchased from Metro Supermarket at Zhenjiang, China. All the samples were stored at -5°C in the refrigerator before experimental process.

The adulterated samples were prepared by blending canola oil with sunflower oil, soybean oil, and peanut oil at levels of 0%, 5%, 10%, 20%, 30%, 40%, and 50% by volume, respectively. The mixed oil samples were brought to room temperature before detection. All the samples (147 samples) were homogenized with vortex for 60s and analyzed immediately after preparation.

### 2.2. Experimental Device

All prepared oil samples were analyzed with GC-IMS device (FlavourSpec®) from G.A.S. (Gesellschaft für Analytische Sensorysteme GmbH, Dortmund, Germany). The instrument was equipped with an incubating device intended to heat the sample and keep the headspace container at a constant temperature and a heated splitless injector for direct sampling of headspace volatile compounds from the oil samples into the GC-IMS. In addition, the device was coupled to an automatic sampler unit (CTC-PAL, CTC Analytics AG, Zwingen, Switzerland), which made the injection volume more accurate and repeatable without any human manipulation. Sample vials were transported into a heated incubator for preconditioning. After they reached equilibrium, a heated gas-tight syringe moved over the incubator and withdrew the headspace sample. After sample injection, the hot syringe was automatically cleaned by purging with inert gas. The experimental parameters used for this method were summarized in Table 1.

For analysis, 2 mL of oil sample was placed in a 10 mL vial which was sealed with a magnetic cap and heated at 90°C for 10 min in incubating box in order to generate volatile compounds from oil sample. 200 *μ*L of headspace was automatically injected by heated syringe (95°C) into the heated injector (95°C) of the GC-IMS instrument. After that, the volatile organic compounds were pushed into the multicapillary column (MCC, 40°C) through a carrier gas (15 mL/min) for timely separation. And then, a drift gas (300 mL/min in counter flow) was generated to collide with the gaseous ions of separated components from the sample to produce the ion mobility spectra at room temperature and pressure, and finally gaseous ions were captured by a detector (a Faraday plate). Data of samples were obtained by IMS Control TFTP Server software and displayed by using LAV software (Version 1.3.1, from G.A.S).

### 2.3. Data Processing

The multidimensional signals of GC-IMS data required pretreatment before statistical analysis. The alignment is an important step before chemometric process of the data due to small deviation in temperature of MCC and flow velocity causing changes in retention time and small deviation in temperature of drift tube causing changes in drift time. Therefore, RIP (Reaction Ion Peak: reaction ions are generated by a cascade of reactions following the collision of a fast electron emitted from ionization source with the drift gas atmosphere; as a consequence, the so-called RIP representing the total of all ions available is formed) normalization was applied to align with a shift in x-axis by LAV software. Moreover, a Savitzky-Golay smoothing method (order 2 and window size 15) was used to standardize the measurement and improve the signal-to-noise rate.

Many studies [27, 28] have proposed various feature extraction methods, such as Gabor filters, local binary pattern, Haar, and histograms of oriented gradients (HOG). HOG is a feature descriptor used in computer vision and image processing for the purpose of object detection. The theory of HOG descriptor is that local object appearance and shape within an image can be described by the distribution of intensity gradients or edge directions. The image is divided into small connected regions called cells, and for the pixels within each cell, a histogram of gradient directions is compiled by counting occurrences of gradient orientation in localized portions of an image. For the present, HOG is proven to offer better feature extraction that could significantly outperform existing feature sets for object detection.

**Figure 1:**
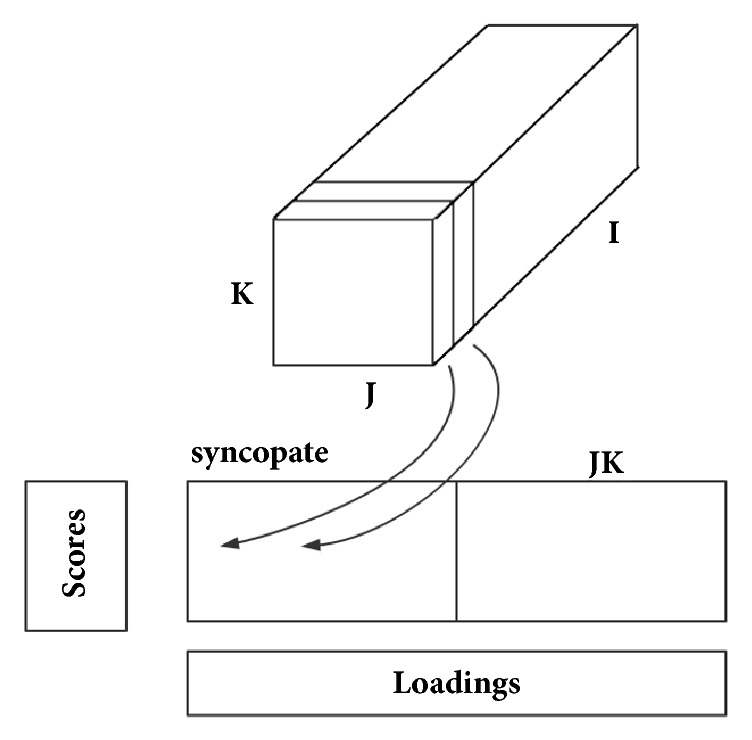
Computation procedure of MPCA method.

Principle component analysis (PCA) is a classical feature extraction and data representation technique widely used in the area of pattern recognition and computer vision [29]. In PCA-based pattern recognition, the 2D (two-dimensional) matrices must be previously transformed into 1D (one-dimensional) vectors. However, the resulting vector usually leads to a high-dimensional vector space, which is difficult to evaluate the covariance matrix accurately due to its large size. Fortunately, a new method, called multiway principal component analysis (MPCA) [30], was developed for matrix feature extraction.  Figure 1 summarizes the computation procedure of MPCA. As mentioned above, each sample was detected by GC-IMS and a 2D matrix was obtained. These sample data are arranged and merged to a three-dimensional matrix  ***X***(*I×J×K*), where * I*  is total samples,  * J*  is drift time, and * K*  is retention time. First, the three-dimensional matrix ***X***  is split along the direction of the sample axis (see Figure 1) and formed a new matrix ***X***(*I×JK*); then, the new matrix ***X***  is decomposed into the product of the score vector ***t***_*r*_  with the load vector ***p***_*r*_, plus the residual matrix ***E***  (see (1)), which is similar to the two-dimensional principal component analysis method.  ***R***  is the number of principal components; ⊗ is the product of Kronecker. As opposed to conventional PCA, MPCA is based on 2D matrices rather than 1D vector. As a result, MPCA has two important advantages over PCA. (1) It is easier to evaluate the covariance matrix accurately. (2) Less time is required to determine the corresponding eigenvectors.

(1) $$\begin{array}{c} X=\overset{R}{\underset{r=1}{\sum }}t_{r}⊗p_{r}^{T}+E \end{array}$$

In our study, a preprocessing step to the statistical analysis of the GC-IMS data was performed to all oil samples which consisted of a RIP normalization and a Savitzky-Golay smoothing filter. Firstly, a HOG method was used to extract texture and contour information. Later, as an unsupervised method, MPCA was applied to further extract the features and visualize the dataset retaining the maximum variability present in the original data and eliminating possible dependence between variables. Then, canonical discriminant analysis (CDA) algorithm was used to generate nonlinear boundaries between classes according to the content of adulterated oil. Lastly, partial least square analysis (PLS) was performed to study the predictive capacity of GC-IMS for the adulteration content of mixed oil.

For data analysis, the MATLAB R2009a (The Mathworks Inc, Natick, USA) and PLS Toolbox 5.5 (Eigenvector Research, Inc., Manson, WA, USA) were used.

## 3. Results and Discussion

### 3.1. Sample Analysis by GC-IMS

**Figure 2:**
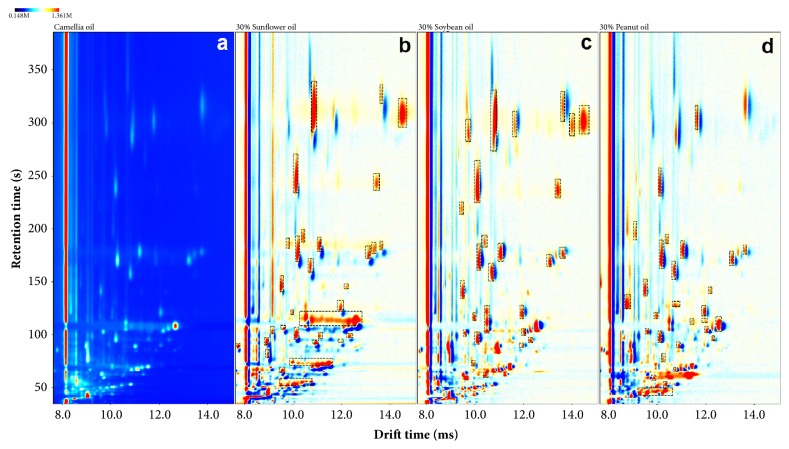
GC-IMS plot comparison of (a) pure canola oil, (b) 30% sunflower oil adulteration, (c) 30% soybean oil adulteration, and (d) 30% peanut oil adulteration.

The size of raw data from each oil sample was huge, so the spectral area was cut (899×1114 dimension) and selected by limiting the retention time from 35.49 to 385.71 s and drift time from 7.666 to 15.086 ms on the basis of retaining the major information. As it is known, GC-IMS spectrum of a sample corresponded to a matrix and could be displayed as a pseudo color image for visualization [31]. A popular method for comparing two matrices is to form a difference image by subtracting the individual element values of one matrix from the corresponding element values of the other matrix. In this condition, a positive difference indicates that the analyzed matrix has a larger element value and a negative difference indicates that the reference matrix has a larger element value. Therefore, taking canola oil sample as reference matrix, the other adulterated samples were regarded as analyzed matrix and colorized differences were formed by subtracting the canola oil sample from the adulterated sample. In this way, with the increment of sunflower oil content, the changes about volatile organic compounds from adulterated samples were clearly visualized.  Figure 2 showed the GC-IMS pseudo color map for change visualization when a raw canola oil sample (Figure 2(a)) was adulterated with 30% sunflower oil (Figure 2(b)), 30% soybean oil (Figure 2(c)), or 30% peanut oil (Figure 2(d)), respectively. The red region indicated that the sample had more volatile components compared with reference sample. The deeper the color, the more components it had and the blue region was the opposite. As shown in Figure 2, there were obvious changes between pure canola oil sample and adulterated canola oil samples. Many new volatile compounds were produced, some peaks were marked by black dotted rectangle for effective observation, and those differences of red regions were reflected in retention time, drift time, and intensity of the corresponding peaks. On the other hand, levels of original volatile compounds in canola oil were weakened by different degree. Those peak differences (drift time, retention time, peak volume, etc.) from volatile organic compounds of each kind of vegetable oil are the key to qualitative analysis or quantitative detection. On the other hand, volatile organic compounds from samples adulterated with soybean oil appeared only in a short retention time, and the gas molecule materials could not be well separated, which may be caused by higher initial carrier gas flow-rate. However, only a general overall impression of the differences through the topographic plot was obtained. The content changes of volatile organic compounds were not regular and it was hard to realize digital characterization. Therefore, further analysis was necessary with the help of chemometric tools.

### 3.2. Adulteration Classification of Oil Samples

**Figure 3:**
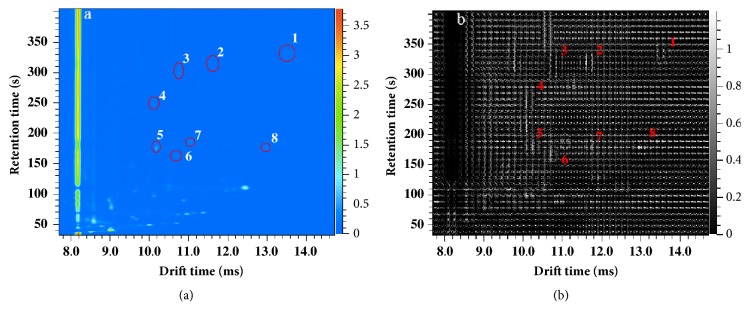
Visualization results of original data (a) and HOG feature extraction (b).

Before MPCA analysis, the data set was processed by HOG algorithm (24×24 block size after optimization) in order to extract useful information and reduce the dimension of the original matrix. As shown in Figure 3, some peaks were selected and marked with red solid line ellipse and Arabic numerals for visual observation in Figure 3(a), which are corresponding to the marked area with the same Arabic numerals in Figure 3(b). It was clear that the visualization of HOG result (Figure 3(b)) could describe the characteristic information of the whole matrix with strong texture structure and contour information from the original data (Figure 3(a)), especially the peaks that were corresponding to volatile organic compounds. In addition, the dimension of the matrix from each sample was effectively reduced (777×966 dimension).

**Figure 4:**
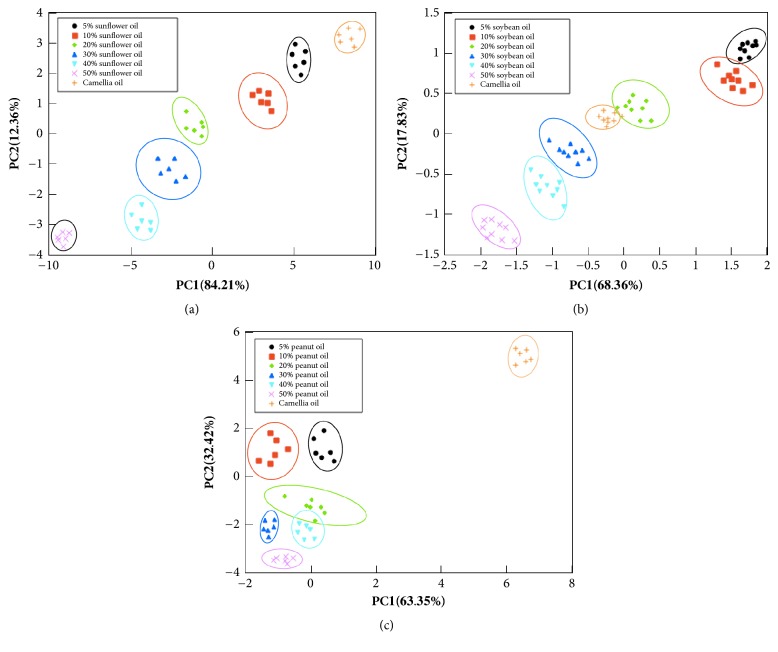
PC1 and PC2 scores of pure canola oil adulterated with (a) sunflower oil, (b) soybean oil, and (c) peanut oil.

As mentioned earlier, each kind of adulterated samples after HOG pretreatment was arranged to a three-dimensional matrix and MPCA algorithm was used to process and analyze the matrix. Principal component scores obtained were sorted from high to low according to the cumulative contribution rate and the first 2 principal component score matrices were used to show the cluster of adulterated samples (see Figure 4). As shown in Figure 4, the data were mapped on two most important principal components PC1 and PC2, presenting the results of canola oil adulterated with sunflower oil (Figure 4(a)), with soybean oil (Figure 4(b)), and with peanut oil (Figure 4(c)). The axis heading in each figure was labeled with the respective contribution rates of PC1 and PC2 after MPCA process.

Figure 4(a) showed the PCA score plot of canola oil adulterated with sunflower oil, and PC1 and PC2 explained the 96.57% of the original information. It could be inferred that the first 2 PCs could give the most information of data set. As shown, the pure canola oil samples were sufficiently well distinguished from adulterated samples with different levels of adulteration and each level of adulteration had its own cluster group. With the increase in the proportion of sunflower oil, the distribution of the samples moved from right to left between first two principal components. Figure 4(b) presented the result of MPCA analysis of the canola oil adulterated with soybean oil samples. Two principal components covered 86.19% of the original information. It was visible that the data points belonging to each class of adulteration rate were gathered in compact clusters and two principal components showed good separation in the direction of diagonal of axis except raw canola oil samples. Although two principal components contained large amount of original information, some clusters overlapped each other (for example, clusters 0% and 20% groups). The results of canola oil containing peanut oil samples were shown in Figure 4(c). The reconstructed information contained 95.77% of the variance. As can be observed, pure canola oil samples and adulterated samples were located in the opposite side of the axis, which indicated that the raw canola oils were well differentiated from adulterated samples. As a result, there were significant differences in aroma between canola oil and peanut oil. Combining with Figure 2(d), it could be inferred that volatile compounds from peanut oil could obviously cover up the original flavor of canola oils. On the other hand, different content of peanut oil also could be distinguished. However, 20% adulterant oil samples clustered in two-dimensional space overlapping with 30% and 40% groups, and its cluster region was a long and narrow strip. This phenomenon may be resulted from the origin differences between peanut oil samples purchased.

Finally, CDA was used as pattern recognition techniques for the authentication of canola oil. As seen in Figure 4, each kind of adulterated oil samples was all grouped into 7 distinct clusters. All canola oil samples adulterated with sunflower oil could be classified without any error (100 % of accuracy). Only 2 samples (one adulterated with soybean oil and the other adulterated with peanut oil) were misclassified. The total accuracy of recognition was 98.64%, which showed excellent results of classification.

### 3.3. Rapid Characterization of Sunflower Oil Content in Canola Oil

In order to establish relationship between the GC-IMS and the content of adulterant oil added to canola oil, an analytical method of partial least square (PLS) was used. Quantification of the percentage of adulterant added to canola oil was carried out by building separate calibration models for each kind of vegetable oil between 0 and 50% level.

The principal component scores of sunflower oil data set were selected as input variables and analyzed by PLS. Before building the model, the samples used for training and testing were randomly selected. Data set containing 70% canola samples was used for calibration and that containing the remaining 30% samples was used to predict the content of sunflower oil adulterated in canola oil. The other two kinds of adulterated samples were treated in the same way. The correlation coefficient (*R*^*2*^) and root mean square error (*RMSE*) between predicted and experimental values were used to evaluate the performance of the model. The higher* R*^*2*^ and lower RMSE mean better calibration model.

PLS is a multivariate projection method for building relationship between dependent variables and independent variables. Due to the limited amount of training samples, leave-one-out cross validation was applied and the first 3 principal components were determined. Correlation coefficient for calibration and prediction (*R*^*2*^*C* and* R*^*2*^*P*) and root mean square error of calibration and prediction (*RMSEC* and* RMSEP*) were tabulated in Table 2; good correlations of calibration were found between GC-IMS data and content of adulterant oil added to canola oil with high coefficient of determination (*R*^*2*^*C*>0.96) and low errors (*RMSEC *≤ 2.92). When PLS models were used to predict the testing data set, good prediction results for the content of adulterant oil added to canola oil were obtained with good coefficient (*R*^*2*^*P*>0.95) and acceptable errors (*RMSEC *≤ 3.23). The* RMSEP* value of sunflower oil adulterated in canola oil is lower than its* RMSEC* value and the possible reason may be that the separation of samples is uneven. The best results were obtained with the model carrying out with the canola oil adulterated with soybean oils. All those models are acceptable and useful for detecting the adulteration in canola oil. Therefore, our present work verifies that adulteration in canola oil can be determined by PLS using the GC-IMS data.

## 4. Conclusions

In this paper, GC-IMS has been proposed to detect the adulteration of canola oil samples with low price vegetable oils. 147 samples were detected and the useful signals were extracted and analyzed by HOG and MPCA algorithms. A combination of these two methods was proved to be the most effective feature extraction method. PLS was used to predict the adulteration levels in canola oil precisely. The capacity in prediction showed that all those methods were satisfactory and applicable. In addition, the model of canola oil adulterated with soybean oil was proved to be the most effective.

The methodology developed has capability to detect adulteration in canola oil. The analysis time only needs about 15 min which is less than other techniques and no sample pretreatment is required. Moreover, the device has been applied in industrial field broadly due to low cost and portability. Therefore, GC-IMS can be seen as a powerful authentication method with chemometrics for oil adulteration detection for its high efficiency and accuracy.

## Figures and Tables

**Table 1:** Experimental conditions for GC-IMS analyses.

	Parameters	Value
Automatic inject system	Headspace sampling volume	200 *μ*L
	Incubation time	10 min
	Sample volume	2 mL
	Incubation temperature	90°C
	Injector temperature	95°C
Column	MCC	OV-5 (nonpolar)
	Column temperature	40°C
	Length of column	30 cm
	Run time	15 min
IMS	Ionization source	Tritium (6.5 KeV)
	Voltage	Positive drift
	Drift length	10 cm
	Carrier gas flow rate	15 mL min^−1^ (N_2_ 5.0)
	Drift gas flow rate	300 mL min^−1^ (N_2_ 5.0)
	Equipment temperature	45°C
	Average	32
	Electric field strength	350 V cm^−1^
	Grid pulse with	100 *μ*s
	Trigger delay	100 ms
	Sampling frequency	150 kHz
	Repetition rate	21 ms

**Table 2:** Requisite parameters for adulteration level prediction in adulterant canola oil samples.

PLS Models	Calibration		Cross validation		Prediction	
	*R* ^*2*^ *C*	*RMSEC*/(%)	*R* ^*2*^ *CV*	*RMSECV*/(%)	*R* ^*2*^ *P*	*RMSEP*/(%)
Adulterated with sunflower oil	0. 994	1.78	0.991	1.80	0.983	1.86
Adulterated with soybean oil	0.996	1.39	0.989	1.43	0.985	1.45
Adulterated with peanut oil	0.968	2.92	0.963	3.18	0.952	3.23

*R* ^*2*^ *C: coefficient of determination for calibration.*

*R* ^*2*^ *CV: coefficient of determination for cross validation.*

*R* ^*2*^ *P: coefficient of determination for prediction.*

*RMSEC: root mean square error of calibration.*

*RMSECV: root mean square error of cross validation.*

*RMSEP: root mean square error of prediction*.

## Data Availability

The data used to support the findings of this study are available from the corresponding author upon request.
